# Evolution and Climate Adaptation in Eurasian Gyrfalcon Populations

**DOI:** 10.1002/ece3.73052

**Published:** 2026-02-10

**Authors:** Xin Liu, Li Hu, Zhenzhen Lin, Shengkai Pan, Siying Huang, Vasiliy Sokolov, Aleksandr Sokolov, Ivan Fufachev, Sergey Ganusevich, Andrew Dixon, Xiangjiang Zhan

**Affiliations:** ^1^ State Key Laboratory of Animal Biodiversity Conservation and Integrated Pest Management Institute of Zoology, Chinese Academy of Sciences Beijing China; ^2^ Key Laboratory of Animal Ecology and Conservation Biology Institute of Zoology, Chinese Academy of Sciences Beijing China; ^3^ Cardiff University–Institute of Zoology Joint Laboratory for Biocomplexity Research, Chinese Academy of Sciences Beijing China; ^4^ University of the Chinese Academy of Sciences Beijing China; ^5^ Yantai Institute of Coastal Zone Research Chinese Academy of Sciences Yantai China; ^6^ College of Life Sciences, China West Normal University Nanchong China; ^7^ Institute of Plant and Animal Ecology, Ural Division Russian Academy of Sciences Ekaterinburg Russia; ^8^ Arctic Research Station of the Institute of Plant and Animal Ecology, Ural Division Russian Academy of Sciences Labytnangi Russia; ^9^ Wild Animal Rescue Centre Moscow Russia; ^10^ Mohamed Bin Zayed Raptor Conservation Fund Abu Dhabi UAE; ^11^ Center for Excellence in Animal Evolution and Genetics Chinese Academy of Sciences Kunming China

**Keywords:** Arctic, climate change, conservation, *Falco rusticolus*, population genomics

## Abstract

Climate change is considered a key driver for shaping ecological and evolutionary processes of Arctic animals. Historical glaciation has profoundly influenced the distribution and genetic differentiation of Arctic vertebrates, and recently Arctic species are facing new and intensifying threats from rapid global warming. Understanding how past, recent and future climate change has, and will influence the evolution of Arctic animals is, therefore, crucial for effective conservation planning. Here we combine whole‐genome sequencing, demographic inference, and species distribution modeling (SDM) to assess the eco‐evolutionary responses of the gyrfalcon ( *Falco rusticolus* ), a resident Arctic apex predator, to climate change. Assembling a genome reference and using samples from three breeding regions across the Eurasian Arctic (Kola, Yamal, and Chukotka peninsulas), we found genetic differentiation of gyrfalcon populations from west to east, that arose during the late Pleistocene (12.9–14.7 thousand years ago (ka)) and subsequently persisted in isolation, until gene flow into the Yamal population resumed during the late Holocene. The extant gyrfalcon populations exhibit low genetic diversity, elevated inbreeding coefficients, and high genetic loads compared to the closely related saker falcon ( *Falco cherrug* ), and some other threatened species with small populations, likely due to a population bottleneck about 1 ka, which might compromise the long‐term viability of this Arctic raptor. Additionally, the effective population size (*N*e) of the Kola gyrfalcon population was inferred to be in decline over the past 165–60 years. SDM based on ensemble models further predicts a substantial reduction of climatically suitable areas for Kola gyrfalcons under future global warming scenarios. Our study highlights how past climatic fluctuations and ongoing warming jointly shape the genomic landscape of endemic Arctic birds and provides insights into making conservation strategies for Arctic animals in a rapidly warming environment.

## Graphical abstract

This reports the influence of past, current and future Arctic climate change to the evolution of Eurasian gyrfalcons (*Falco rusticolus*), and reveals that its current evolutionary potential is insufficient to cope with the ongoing Arctic warming.

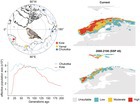

## 1 Introduction

Rapid climate change is reshaping ecological and evolutionary processes across the globe, with Arctic ecosystems among the most severely affected (Gilg et al. 2012). Quaternary glacial–interglacial cycles have shaped the distribution and genetic differentiation of Arctic vertebrates (Hewitt 2000; Hewitt 2004; Fu and Wen 2023). During glacial maxima, populations were frequently fragmented and geographically isolated mainly due to advanced ice sheets, leading to population divergence, even local extinction (Garg et al. 2020). When ice sheets retreated and new habitats became available, population colonization proceeded typically via leading‐edge expansion (Hewitt 1999). Cyclical climatic processes have left signatures on the genomes of Arctic animals through repeated founder events that result in ancestral allele loss, new mutation generation, and ultimately, genetic differentiation (Klopfstein et al. 2005; Schulte et al. 2013). Furthermore, increased habitat connectivity facilitated gene flow between previously isolated populations, allowing them to exchange genetic materials and acquire novel genetic variants (Cramer et al. 2023; Bustillo‐de la Rosa et al. 2024). For example, postglacial secondary contact between previously isolated populations of dunlins ( *Calidris alpina* ) was found to promote gene flow that has contributed to the spread of locally adaptive traits (Wenink et al. 1996).

In contrast to the long‐term climatic fluctuations associated with glacial–interglacial cycles, recent anthropogenic climate change has led to pronounced Arctic amplification. There is an amplification effect with warming in the Arctic being nearly four times faster than the global average since 1979 (Rantanen et al. 2022). Rising temperature has resulted in widespread habitat change, reduced prey availability, and disruptions of food‐webs related to many Arctic vertebrates, while simultaneously facilitating the northward expansion of some Arctic and non‐Arctic animals (Kuletz et al. 2024). For example, red fox ( *Vulpes vulpes* ) has expanded its distribution range into the Arctic tundra, resulting in competition with the native Arctic fox ( *V. lagopus* ) for food and territory (Elmhagen et al. 2017). In southern parts of the Yamal peninsula, lemming occurrence has declined, whereas vole populations have increased and expanded northwards (Sokolova et al. 2024). Moreover, the population of most Arctic‐breeding waders (Charadriiformes) are currently declining (Smith et al. 2020) due to climate‐driven habitat degradation and phenological mismatches (Kwon et al. 2019). Assessing historical and current demography of Arctic species and predicting their response to future climate change is, therefore, essential for developing effective conservation strategies.

The gyrfalcon, a resident raptorial bird with a Holarctic distribution exclusively across Arctic and sub‐Arctic regions, serves as an ideal model for addressing this issue. The breeding distribution ranges of gyrfalcons have experienced repeated expansion, contraction, and fragmentation during Pleistocene glaciations, while recent anthropogenic climate warming poses new and unprecedented challenges (Cade 2011). Previous studies suggested that the primary prey resources for gyrfalcon—willow ptarmigan ( *Lagopus lagopus* ), rock ptarmigan ( *L. muta* ), and Arctic ground squirrels ( *Urocitellus parryii* ) have been decreasing in parts of the Arctic. For example, willow ptarmigan in Fennoscandia and eastern Russia (Lehikoinen et al. 2014; Fuglei et al. 2020), rock ptarmigan in Iceland, Greenland, and mainland Europe (Imperio et al. 2013; Furrer et al. 2016; Canonne et al. 2020), and Arctic ground squirrels at high elevations (Werner et al. 2015) were reported to have declining trends in recent decades. In addition, there is an example from Greenland, where peregrine falcons ( *Falco peregrinus* ) have expanded northward into previously unoccupied Arctic areas, which has potentially intensified interspecific competition with gyrfalcons (Burnham et al. 2012). Similar northward expansion of golden eagles ( *Aquila chrysaetos* ) in parts of northern Fennoscandia (e.g., Finnmark, northern Norway) results in direct competition for nest sites (Johansen and Østlyngen 2011).

In this study, we assembled a gyrfalcon reference genome and analyzed whole‐genome sequences from 26 wild gyrfalcons collected from three breeding regions across Eurasian Arctic (i.e., Kola, Yamal, and Chukotka peninsulas), together with seven saker falcon genomes from a European population (Hu et al. 2022). We utilized population genomic data to understand the evolutionary trajectories of Eurasian gyrfalcon populations from the late Pleistocene to present and to predict their response to future climate change. Specifically, this study aims to (1) examine how past climatic fluctuations have shaped the genetic landscape of Eurasian gyrfalcons by analyzing population structure and demographic history; (2) quantify the long‐term genetic consequences of bottlenecks by estimating accumulated genetic load in contemporary populations; and (3) evaluate how past population bottlenecks may continue to impact the species' evolutionary potential. By integrating historically east–west population structure with climate‐driven north–south changes in population range and habitat suitability, we aim to disentangle how past and contemporary climate changes jointly shape current gyrfalcon populations. Our findings offer important insights into the impacts of ongoing environmental change and provide valuable information for conservation management.

## 2 Materials and Methods

### 2.1 Ethics Oversight

All laboratory experimental procedures were under the guidance of the Ethics Committee of the Institute of Zoology, Chinese Academy of Sciences (No. IOZ‐IACUC‐2021‐101). The collection and processing of gyrfalcon blood samples in this study were conducted in accordance with the guidelines of the Institutional Animal Care and Use Committee of the Institute of Zoology, Chinese Academy of Sciences.

### 2.2 Sample Collection and Whole‐Genome Sequencing

We sequenced 18 wild gyrfalcons (one individual per nest) collected from nestlings in three breeding regions across the Eurasian Arctic: Yamal, Kola, and Chukotka (Table S1). Genomic DNA was extracted using the protocol described by Zhan et al. (2013). Briefly, genomic DNA was extracted using the Blood/Tissue Genomic DNA Kit (QIAGEN), and whole genome resequencing was conducted on a BGISEQ‐500 platform (BGI). We also obtained genome resequencing data of eight gyrfalcons and seven European sakers from our previous study (Hu et al. 2022). European saker falcon was used as an outgroup for subsequent population genomic studies since it is a closely related species (Hu et al. 2022).

### 2.3 Reference Genome Assembly and Annotation

For genome assembly, we extracted genomic DNA from blood of a wild‐origin adult gyrfalcon from Russia that was confiscated by authorities and kept in Abu Dhabi, UAE. We constructed DNA libraries with insert sizes of 170 bp, 500 bp and 800 bp, 5 kb, 10 kb and 20 kb as previously described (Zhan et al. 2013) and subjected to sequencing on an Illumina platform. The generated raw reads were filtered as follows: Reads with > 10% ambiguous bases; reads with low‐quality bases (≥ 40% bases with Illumina *Q*‐value ≤ 7 for the reads from short insert‐size libraries, ≥ 60% bases with *Q* ≤ 7 for the reads from long insert‐size libraries); reads with adapter sequence contamination; short insert reads when the two reads for the paired ends overlapped ≥ 10 bp and the mismatch was < 10%; PCR duplications. We finally obtained a total of 147 Gb clean data for genome assembly (Table S2). *SOAPdenovo* (Li et al. 2010) was employed for draft genome assembly from the second‐generation sequencing method. Then, 12.56 Gb data generated from third‐generation sequencing method (PacBio) (Table S2) were corrected using LoRDEC (Salmela and Rivals 2014) with the short insert size sequencing reads, and assistance for super‐scaffolds assembly using *SSPACE‐LongRead* with default parameters (Boetzer and Pirovano 2014).

Genes were annotated following our previous pipeline (Zhan et al. 2013). Briefly, genomic repetitive sequences were firstly annotated using RepeatMasker (Tarailo‐Graovac and Chen 2009). For homolog gene prediction, we mapped the protein sequences of *Gallus gallus* , *Taeniopygia guttata* , *Falco peregrinus* and *Falco cherrug* , and *Falco rusticolus* against the assembled genome using TBLASTN (version 2.2.23) with an *E*‐value threshold of 1E‐5 and determined gene models using GENEWISE (Birney et al. 2004) (v2.2.0). For the *de novo* predictions, we trained the parameters using the well annotated genes from homolog evidence. The trained parameters were used to predict candidate genes using AUGUSTUS (Stanke and Morgenstern 2005) (version 3.3.2) and SNAP (Korf 2004). For the transcriptome‐based prediction, we aligned the gyrfalcon blood transcriptome data (Zuccolo et al. 2023) with the assembled genome using HISAT2 (Kim et al. 2019) and identified transcripts using StringTie (Pertea et al. 2015). Genes were finally integrated using EVM (Haas et al. 2008). Function annotations were conducted by aligning each protein sequence to SwissProt and TrEMBL databases using BlastP.

### 2.4 Data Filtering and SNP Calling

Sequenced raw reads were quality‐filtered using *fastp* version 0.21.0 (Chen et al. 2018), and mapped to the gyrfalcon reference genome using BWA‐MEM version 0.78 (Li 2013). Samtools version 1.19 (Danecek et al. 2021) and Picard Tools version 1.56 (http://broadinstitute.github.io/picard) were used to sort bam files and filter duplicate reads. Variant calling was performed using the command HaplotypeCaller and GenotypeGVCFs of the GATK (Genome Analysis ToolKit package) version 4.0.7 (McKenna et al. 2010), and then were filtered using the VariantFiltration command with the following criteria: “(1/3 sum Depth) || DP > (3 sum Depth); QD < 2.0; FS > 60.0; MQ < 40.0; MQRankSum < −12.5; ReadPosRankSum < −8.0; SOR > 3.0”. High‐quality SNPs with missing rate < 30% were retained. Autosomal SNPs were obtained by excluding variants from super‐scaffolds aligned to the Z and W chromosomes of the saker falcon reference genome (Hu et al. 2022), as determined by MUMmer (version 4.0.0) (Marçais et al. 2018) (Figure S1).

### 2.5 Population Structure and Genetic Differentiation

We identified kinship among the 26 gyrfalcons (unknown pedigree) before population structure analysis to control relatedness bias. The kinship coefficient of each pair of individuals was calculated using KING version 2.2.5 (Manichaikul et al. 2010) based on our population genomic SNP data. For pairs of individuals with a kinship coefficient > 0.0884, one member was randomly excluded. We excluded the C18 sample for population analysis due to a close relation with the C14 (Figure S2).

Based on autosomal SNPs located in annotated non‐genic regions, we used two methods to explore potential population structure within the studied gyrfalcon populations. ADMIXTURE version 1.3.0 (Wang 2022) was implemented to infer population ancestry components, running with the group number *K* set as 1–5 and bootstrapping 100 replicates. We also conducted a principal component analysis (PCA) using PLINK version 1.9 (Purcell et al. 2007).

To quantify population genetic differentiation, we calculated the pairwise absolute genetic divergence (*d*_XY_) (Fu et al. 2022) across the genome with 10‐kb windows for three pairwise population comparisons: Yamal vs. Kola, Yamal vs. Chukotka, and Kola vs. Chukotka using the popgenWindows.py script (https://github.com/simonhmartin/genomics_general). The SNPs data without filtering MAF were used for *d*_XY_ calculation.

### 2.6 Phylogenetic Reconstruction and Gene Flow Estimation

We reconstructed phylogenetic relationships among gyrfalcon individuals using two complementary approaches, with the saker falcon as an outgroup. First, we constructed a maximum likelihood tree based on 195,655 autosomal SNPs, which were thinned using a 3‐kb window size to minimize linkage disequilibrium (LD), resulting in the removal of 1,714,886 SNPs. Tree inference was performed using FastTree with default settings (Price et al. 2009). Second, we performed MP‐EST analysis (Liu et al. 2010) based on all 100‐kb window sequences to infer tree topologies. To shorten the computation time efficiently, one individual with high sequencing coverage (> 20‐fold) from each population (including gyrfalcons from Chukotka, Yamal, Kola, and European saker populations) was used as representatives. We then mapped the sequencing reads of these four individuals with the gyrfalcon genome assembly using BWA‐MEM and extracted the genomic consensus sequences from BAM files using BCFtools (Danecek et al. 2021). Gene trees were inferred for each window using RAxML under the GTRGAMMAI model with 100 bootstrap replicates (Stamatakis 2014). A total of 9513 gene trees derived from 100‐kb windows were used as input for MP‐EST analyses. Gene trees inferred from 50‐kb and 200‐kb windows were used to assess the robustness of phylogenetic inference to window size.

Both Dsuite (Malinsky et al. 2021) and QuIBL (Edelman et al. 2019) were used to detect potential introgressions among gyrfalcon populations. Patterson's *D* statistic (ABBA‐BABA test) (Green et al. 2010) was computed using Dsuite. The (((P1, P2), P3), O) topology was set as (((Yamal, Kola), Chukotka), saker). Statistical significance was assessed via block jackknifing, and *Z*‐scores greater than three were considered indicative of genomic introgression.

To further investigate gene flow between the Yamal and the other two populations, we applied QuIBL (Edelman et al. 2019), a branch length‐based method (quantifying introgression via branch lengths). A total of 190,265 maximum likelihood (ML) trees were reconstructed using non‐overlapping 5‐kb windows across the genome. To reduce computational load while focusing on the triplet involving the Yamal, Kola, and Chukotka populations, we randomly subsampled 9400 local ML trees. In each run, Bayesian information criterion (BIC) scores were calculated for two competing models (BIC1 for incomplete lineage sorting (ILS) only model, and BIC2 for ILS with introgression model) under each of the three alternative topologies. Model preference was determined based on the difference in BIC values (*Δ*BIC = BIC2—BIC1): *Δ*BIC < −10 supports the ILS with introgression model, while *Δ*BIC > 10 supports the alternative (Feng et al. 2022).

### 2.7 Demographic History Inference

We inferred ancient and recent demographic histories of three gyrfalcon populations using different methods. The *N*e changes from two million years ago to 20 ka were estimated using PSMC version 0.6.5‐r67 (Li and Durbin 2011) with parameters “‐*N*25, *t*15, ‐*r*5, ‐*p* 4 + 25*2 + 4 + 6”. We also tuned the parameter *‐p* for PSMC estimation to assess its sensitivity (Figure S3), since *‐p* directly controls the discretization of time intervals and has the strongest influence on the shape and resolution of inferred *N*e trajectories. The *N*e changes from 30 ka to 1 ka were evaluated using PopsizeABC (Boitard et al. 2016). The *N*e trend over the past 1.3 ka was reconstructed using GONE (Novo, Ordás, et al. 2023). Mutation rate was assumed as 1.1 × 10^−8^ per generation and generation time was 6.6 years in all the methods (Hu et al. 2022).

We also performed coalescent simulations using fastsimcoal2 (Excoffier et al. 2021) based on the composite likelihood method to reconstruct a scenario of population divergence and gene flow among gyrfalcon populations. Folded multidimensional site frequency spectrum was generated using EasySFS (Gutenkunst et al. 2009), and 18 historic models among gyrfalcon populations were simulated (Figure S4 and Table S9). For each model, we performed 100 independent runs and obtained the corresponding maximum likelihood values with parameters “*‐n* 100,000, *‐N* 100,000”. The best‐fitting model was selected based on the lowest Akaike Information Criterion (AIC) value. Finally, the best‐fit model was run with 100 bootstraps to compute parameter confidence intervals.

### 2.8 Estimation of Genome‐Wide Heterozygosity, Inbreeding Coefficient and Genetic Load

We estimated genome‐wide heterozygosity using PLINK (version 1.9) with the –het parameter, and identified runs of homozygosity (ROH) with the following parameters: –*homozyg –homozyg‐window‐snp* 50 –homozyg‐snp 50 *–homozyg‐kb* 100. The SNPs without MAF filtering were used to identify ROH. The genomic inbreeding coefficient was calculated as the total length of ROHs divided by callable genome length (FROH). Genome‐wide heterozygosity was evaluated from the proportion of heterozygous sites in the genome. We assessed LD decay for each gyrfalcon population using PopLDdecay (Zhang et al. 2018).

Furthermore, we identified deleterious mutations including synonymous, missense and loss‐of‐function (LOF) variants located in coding regions for each gyrfalcon and saker population using SnpEff version 5.2 (Cingolani et al. 2012). Missense variants were considered deleterious if Grantham's score (Grantham 1974) was greater than 150; others were considered benign variants (Feng et al. 2019). We defined ancestral alleles as those shared between gyrfalcon and saker falcon, ignoring sites that were heterozygotes for the same two alleles in both genomes (Hu et al. 2020; Gates et al. 2025). The mutation burden of homozygous‐derived alleles under evolutionary constraint is used as an indicator of genetic load and serves as a proxy for their potential impact on fitness (Marsden et al. 2016; Feng et al. 2019; van der Valk et al. 2019). We used KOBAS (Bu et al. 2021) to enrich KEGG pathways of the identified LOF genes. Pathways with significant corrected *p* values (< 0.05) were displayed.

### 2.9 Genomic Selection Signatures Between Kola and Chukotka Populations

We scanned the genomic regions under selection between Kola and Chukotka populations using the XP‐EHH method implemented in selscan (Szpiech and Hernandez 2014). XP‐EHH scores were calculated in 10‐kb sliding windows. The top 1% and bottom 1% of windows were considered as candidate regions under positive selection in the Kola and Chukotka populations, respectively (Sabeti et al. 2007).

### 2.10 Prediction of Future *N*e Fluctuations

We forecast the *N*e trend under future climate change using our published pipeline (Gu et al. 2021). Briefly, we firstly reconstructed *N*e changes over the most recent 200 generations using GONE (Pérez‐Pereira, et al. 2023), and estimated the *N*e change trends per generation for each population using *N*_e_S (*N*e slope) (Pitt et al. 2019) since the Industrial Revolution (about 9–18 generations for gyrfalcons).

For historical climate data, we downloaded gridded daily data from the Berkeley Earth Surface Temperature dataset (Rohde and Hausfather 2020). Because April and May are the key periods for egg‐laying and incubation in Eurasian gyrfalcons (Sokolov et al. 2017), we calculated three annual parameters (including mean temperature, the number of extreme high‐temperature days, and the number of extreme low‐temperature days) of these 2 months for estimation. To quantify historical annual temperature extremes, the extreme high‐temperature days were defined as days when the daily maximum temperature exceeded the 95th percentile, while extreme low‐temperature days were defined as days when the daily minimum temperature fell below the 5th percentile.

Then we investigated the association between *N*_e_S and historical climate variables (including annual mean temperature, the frequency of extreme high‐temperature days, and the frequency of extreme low‐temperature days during critical breeding months) using a generalized linear model (GLM). The GLM quantified the influence of temporal trends in key climate variables on *NeS*.

To study how *N*e will change under future climate change, we obtained the future daily temperature projection data (2028–2100) from the NASA Earth Exchange Global Daily Downscaled Projections (Thrasher et al. 2022). We used the same percentile‐based approach as historical extremes to infer future weather extremes. Using the fitted model, we finally predicted *N*_e_S under future climate warming from 2028 to 2100. We note that the *N*_e_S is always used to predict the *N*e trend not the real *N*e. We didn't show the analysis results of the Yamal population owing to a biased estimation, probably from gene flow (Novo, Pérez‐Pereira, et al. 2023) (see Results).

### 2.11 Species Distribution Modeling Simulation

We employed biomod2 (Maya et al. 2024) to predict potential breeding areas for Kola gyrfalcons. Briefly, 19 bioclimatic variables (bio01‐bio19) (Table S3) with 10 arc‐min spatial resolution from the WorldClim database (version 2.0) were initially selected for the current (1970–2000) and future (2080–2100) climate conditions under the SSP245 and SSP585 scenarios (Fick and Hijmans 2017). Gyrfalcon occurrence records were compiled from the GBIF (https://www.gbif.org/), for which sufficient records existed only for the Kola region, encompassing Fennoscandia and Lapland of western continental Eurasia. To minimize the effects of over‐sampling, we applied spatial thinning and retained those with an occurrence distance > 50 km; *N* = 139 (Aiello‐Lammens et al. 2015). We generated two sets of 1000 pseudo‐ absence points from background points using the ‘random’ command. Model parameters were optimized with the ‘Bigboss’ command. We used 80% of occurrence data for model training and 20% for validation. Before building the model, we first computed Spearman correlations among the 19 environmental variables to reduce the risk of overfitting. Only environmental variables with correlation coefficients |*r*| < 0.8 were retained (Tables S4 and S5). During simulation, we implemented an ensemble modeling framework that integrates a diverse set of predictive algorithms, including Artificial Neural Network, Generalized Boosting Model, Generalized Linear Model, Multiple Adaptive Regression Splines, Maximum Entropy, Random Forest and Random Forest downsampled, eXtreme Gradient Boosting Training. Final ensemble predictions were projected to climate scenarios from 2080 to 2100.

## 3 Results

### 3.1 Population Structure of Eurasian Gyrfalcons

**FIGURE 1:**
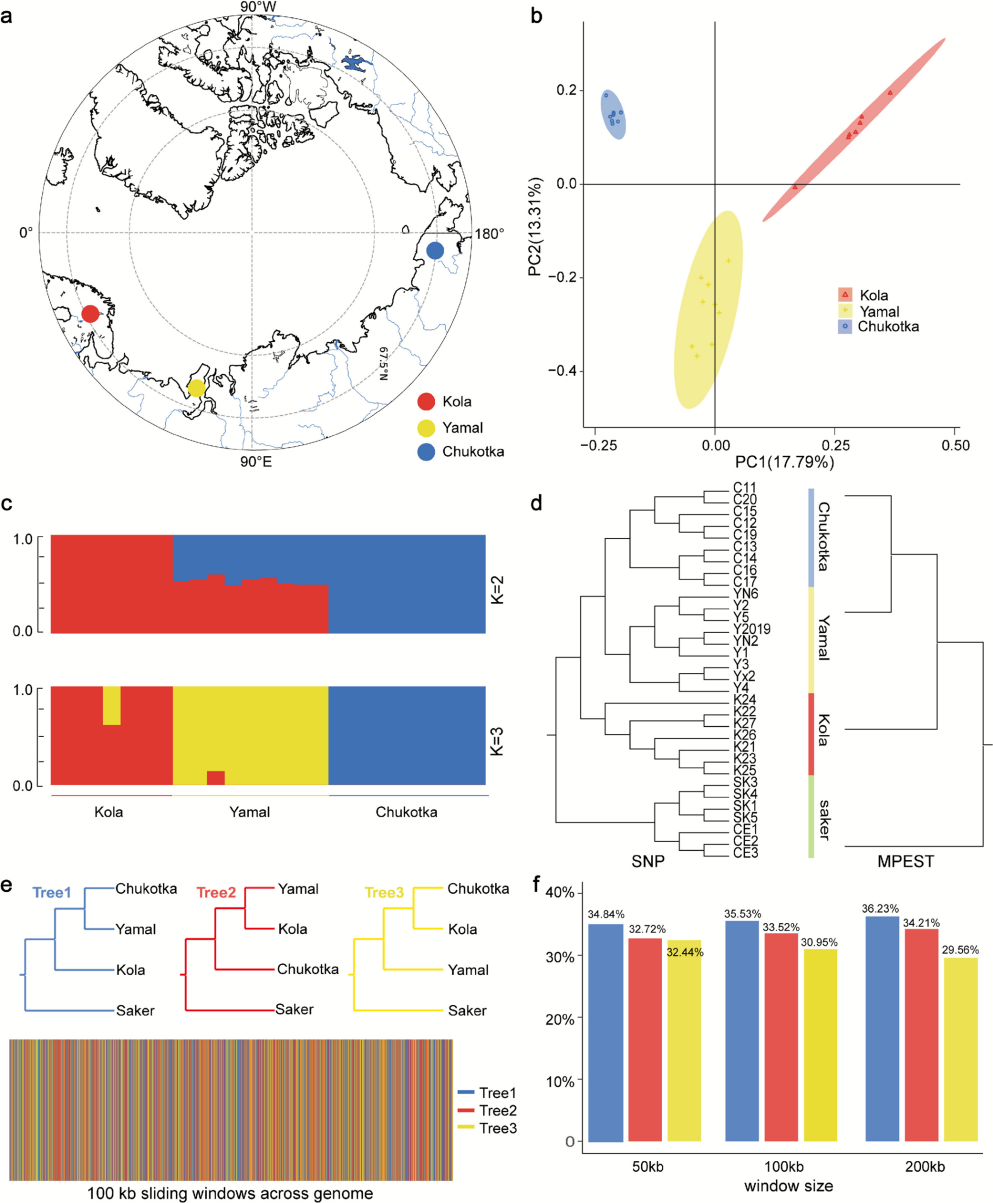
Population genetic structure and phylogenies. (a) Sampling localities for the 26 wild gyrfalcon individuals. (b) Principal component analysis results identified with autosomal nongenic SNPs. (c) Admixture results of the studied gyrfalcons (*K* = 2 or 3). (d) Phylogenetic tree of gyrfalcon individuals constructed using MP‐EST and Fasttree. Both analyses produced the same topology, with strong support (100%) for all interspecific‐level branches. Saker falcons were used as outgroup. (e) Distributions of the phylogenetic tree topologies (Tree‐1, Tree‐2, Tree‐3) across the gyrfalcon genome based on 100‐kb sliding window. (f) Proportion of three phylogenetic trees with different sliding window sizes (50, 100, and 200 kb, respectively).

We have assembled a gyrfalcon reference genome (1.22 Gb, 1444 scaffolds > 2 Kb; scaffold N50, 28.06 Mb) (Table S6) and obtained genomics data from 26 wild gyrfalcons from three breeding areas across the Eurasian Arctic, comprising 9 individuals from Yamal, 7 from Kola, and 10 from Chukotka (Figure 1a). Over 25 Gb of data for each sample were obtained after filtering (Table S1), and the alignment rates to the reference genome were all greater than 90% (Table S1). A total of 2.94 million autosomal SNPs were called using the GATK pipeline after filtering.

Our PCA results showed that Kola, Yamal, and Chukotka gyrfalcons were clustered into distinct genetic populations, with 17.8% of variance explained by principal component 1 (PC1) (Figure 1b). ADMIXTURE analysis reiterated a clear divergence between Kola and Chukotka populations and a mixed background in Yamal individuals (the optimal *K* = 2; Figure 1c and Figure S5), followed by clear divergence among the three populations when *K* = 3 (Figure 1c). The results indicated that gene flow might exist from either Kola or Chukotka to Yamal.

### 3.2 Phylogenetic Conflict Across Gyrfalcon Genomes

We have used MP‐EST and FASTtree to reconstruct the phylogeny of three gyrfalcon populations and converged on the same topologies in which all the gyrfalcons were divided into two groups.

The phylogenetic reconstruction revealed that the Kola individuals formed a basal group, while the Yamal and Chukotka individuals formed distinct clades that were sister to one another (Figure 1d). However, when we respectively examined the distribution of alternative phylogenetic topologies across the genome using sliding windows of 50, 100, and 200 kb, we found that although the MP‐EST/FastTree topology (Tree‐1) occurred the most frequently, it just slightly exceeded the proportions of the two alternative topologies (Tree‐2 and 3). Tree‐1 accounted for 34.84%, 35.53%, and 36.23% of windows at the 50, 100, and 200 kb scales, respectively, in comparison with 32.72%, 33.52%, 34.21% for Tree‐2 and 32.44%, 30.95%, 29.56% for Tree‐3 (Figure 1e,f). The near‐equal representation of the three topologies suggested a high degree of phylogenetic discordance across the gyrfalcon genome, likely resulting from rapid population divergence and incomplete lineage sorting. Consistent with this interpretation, we observed low and comparable levels of genetic differentiation among populations based on pairwise *d*_XY_ index (Kola‐Yamal: 0.000594; Chukotka‐Yamal: 0.000585; Kola‐Chukotka: 0.000603) (Figure S6), supporting a rapid population divergence among these three Eurasian populations.

### 3.3 Genomic Introgression Signals in the Yamal Population

**FIGURE 2:**
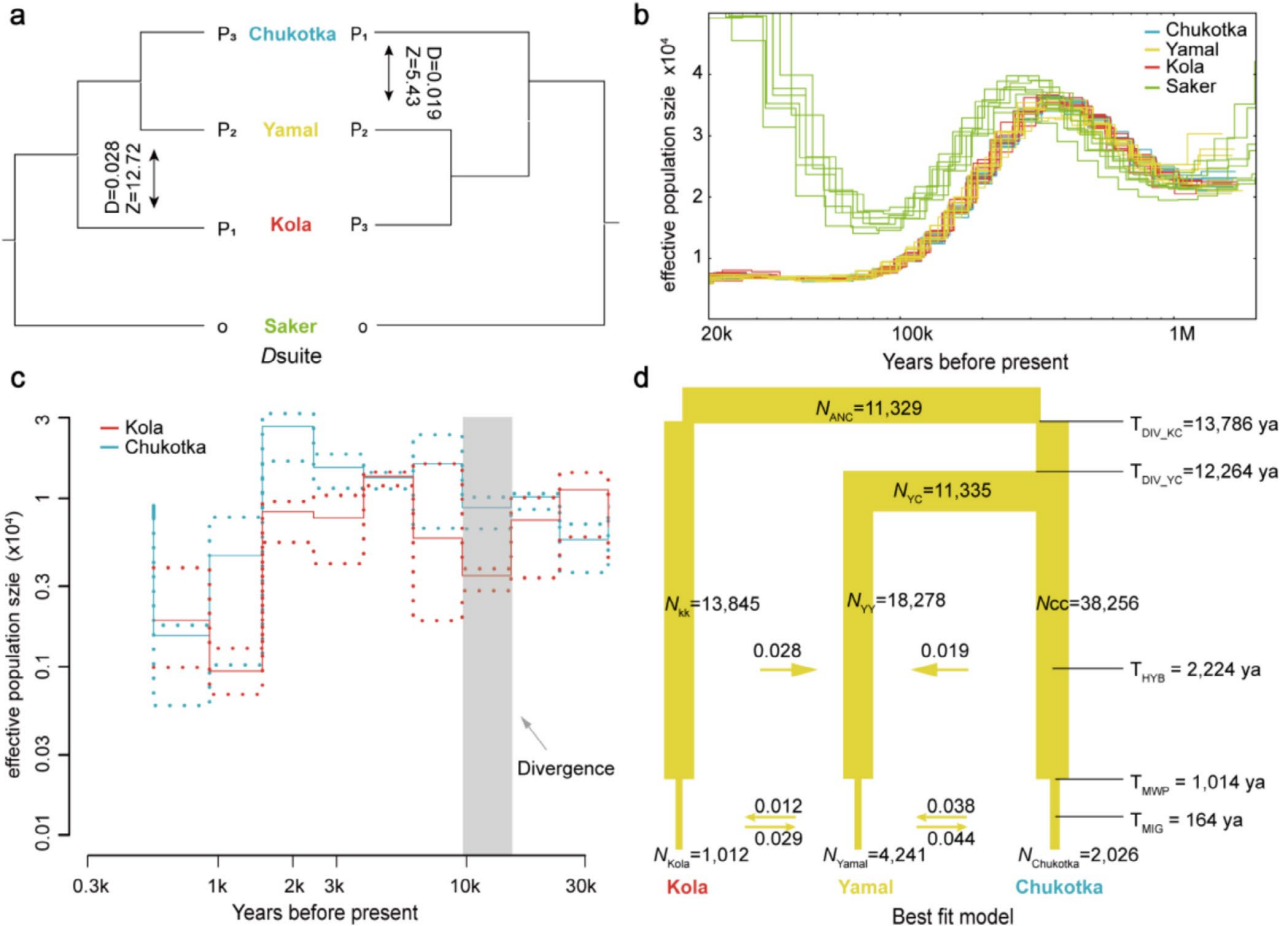
Demographic history reconstruction of Eurasian gyrfalcon populations. (a) Gene flow between Yamal population and the two putative parental populations calculated using *D*‐statistics. (b, c) Population demography of studied gyrfalcons reconstructed using PSMC and PopsizeABC (μ = 1.1*10^−8^, g = 6.6 years) respectively. (d) The best fitting model of population demography simulated using fastsimocal2. *N* means the estimated effective population size. T means years before present (ya). Numbers on the arrows represent estimated gene flow rate.

We used the ABBA‐BABA test to infer whether the Yamal population received alleles from the western Kola or eastern Chukotka populations. As expected, *D*‐statistics analyses revealed significant excess allele sharing between Yamal and both western and eastern populations. The test *D*_(((Chukotka, Yamal), Kola), saker)_ yielded a value of 0.028 (*Z* = 12.4), and *D*_(((Kola, Yamal), Chukotka), saker)_ was 0.019 (*Z* = 5.4) (Figure 2a), with both values significantly different from zero and *Z* > 3 (Table S7), showing gene flow signatures between the Yamal population and the other two populations. Our QuIBL analysis further indicated a “ILS with introgression” model in the formation of the Yamal population (*Δ*BIC < −10; Table S8).

### 3.4 Demographic Histories of Eurasian Gyrfalcons

We reconstructed the demographic histories of Eurasian gyrfalcons using PSMC and PopsizeABC. The PSMC results showed that the *N*e of gyrfalcons increased between one million years and 300 ka. Subsequently, the *N*e of gyrfalcon population sharply decreased (ancient bottleneck) between 300 and 100 ka, whereupon it stabilized after the Last Glacial Period (Figure 2b). The three gyrfalcon populations shared the same *N*e trends in PSMC reconstruction (Figure 2b), showing no divergence among them until 20 ka. The PopsizeABC analyses then inferred that the Chukotka and Kola populations diverged approximately 14 ka (Figure 2c). However, both populations underwent recent bottlenecks in about 1.0 ka during the Medieval Warm Period (Figure 2c).

In addition, we used coalescent simulations implemented in fastsimcoal2 to infer the most likely evolutionary scenario for divergence of Eurasian gyrfalcons. The best‐fitting model supported the divergence with historical gene flow model, i.e., the three gyrfalcon populations diverged during the late Pleistocene (CI: 13,711–13,861) (Figure 2d and Table S9), with subsequent gene flow from the Kola and Chukotka populations to the Yamal population approximately 1.94 ka (CI: 2197–2244) during the late Holocene (Figure 2d and Table S10).

### 3.5 Genetic Diversity and Genetic Load Levels of Eurasian Gyrfalcons

We compared the genetic diversity level of three Eurasian gyrfalcon populations with that of the saker falcon.

**FIGURE 3:**
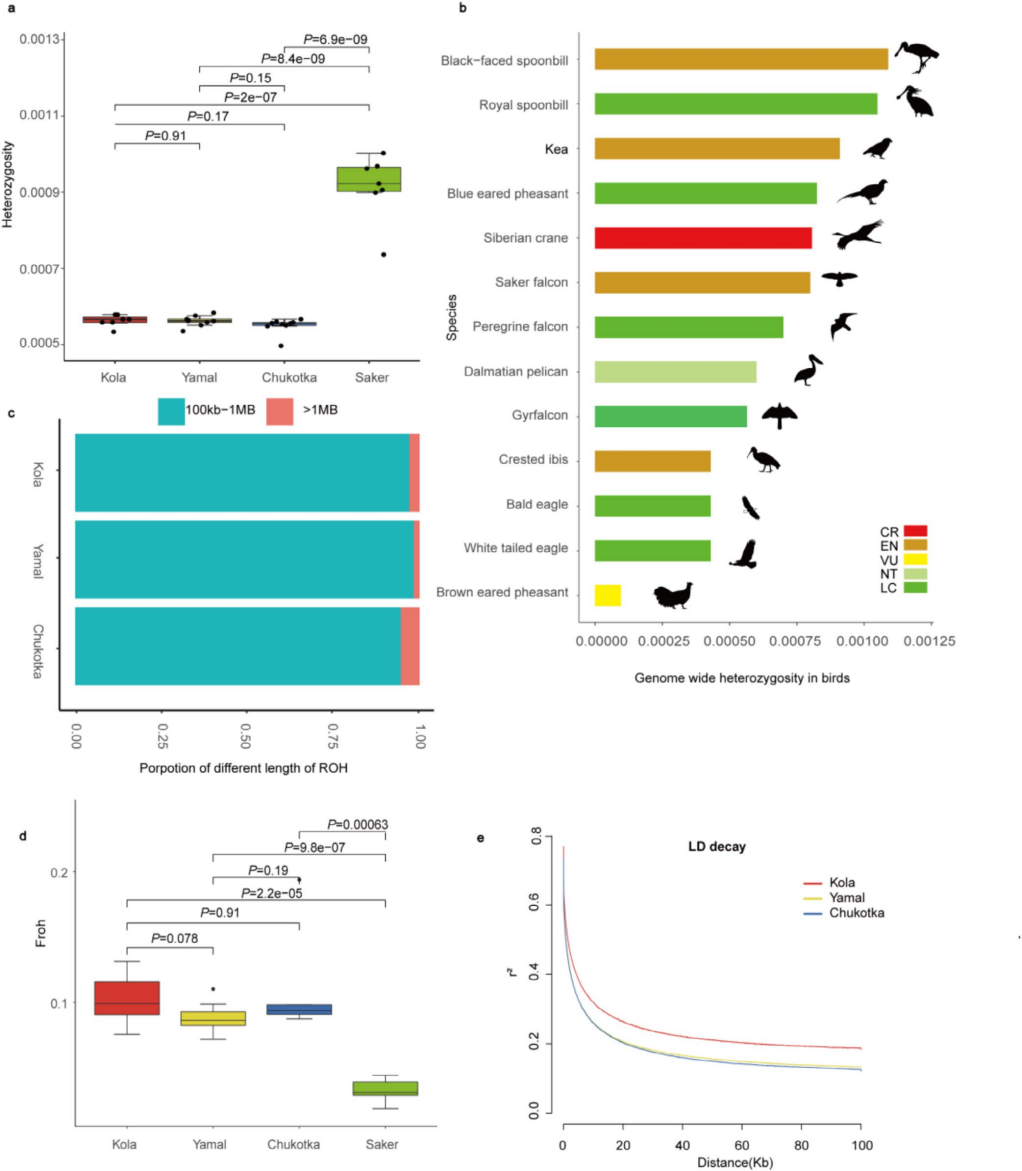
Genetic diversity and inbreeding comparisons among three gyrfalcon populations. (a) Genomic heterozygosity estimation for different gyrfalcon populations. (b) Genomic heterozygosity estimation of 13 representative birds. (c) Proportion of different lengths of runs of homozygosity (ROHs). The blue and red colors represent short (0.1–1 Mb) and long (≥ 1 Mb) ROHs respectively. (d–e) Inbreeding coefficient based on run of homozygosity (FROH) and linkage disequilibrium (LD) decay of three gyrfalcon populations.

Genomic heterozygosity between the Kola, Yamal, and Chukotka populations was comparable (Figure 3a and Table S11), but the genomic heterozygosity of these gyrfalcon (IUCN Red List: Least Concern (LC)) populations was significantly lower than the saker falcon (Endangered (EN); *p* = 2.0e‐7) (Figure 3a and Table S11). In addition, when comparing the genomic heterozygosity of Eurasian gyrfalcons with those of 12 bird species (Table S12), we found that gyrfalcons exhibit significantly lower levels of heterozygosity than eight of them, including three threatened birds: Siberian crane (*Leucogeranus leucogeranus*; Critically Endangered (CR)) (Chen et al. 2025), Dalmatian pelican ( *Pelecanus crispus* ; Near Threatened (NT)), and Kea ( *Nestor notabilis* ; EN) (Li et al. 2014) (Figure 3b and Table S12).

The recent inbreeding level, quantified as the proportion of the genome contained within ROH segments > 1 Mb, was highest in the Chukotka population (5.5%), followed by Kola (3.0%) and Yamal (1.7%; Figure 3c). The genomic inbreeding coefficient FROH of the three gyrfalcon populations is comparable (Kola: 10.27%; Yamal: 8.81%; Chukotka: 10.43%), but much higher than that of sakers (3.24%) or Siberian cranes (4.36%) (Chen et al. 2025) (Figure 3d and Table S13). Within gyrfalcon populations, LD was relatively high in the Kola population, but lower in the Yamal and Chukotka populations (Figure 3e).

**FIGURE 4:**
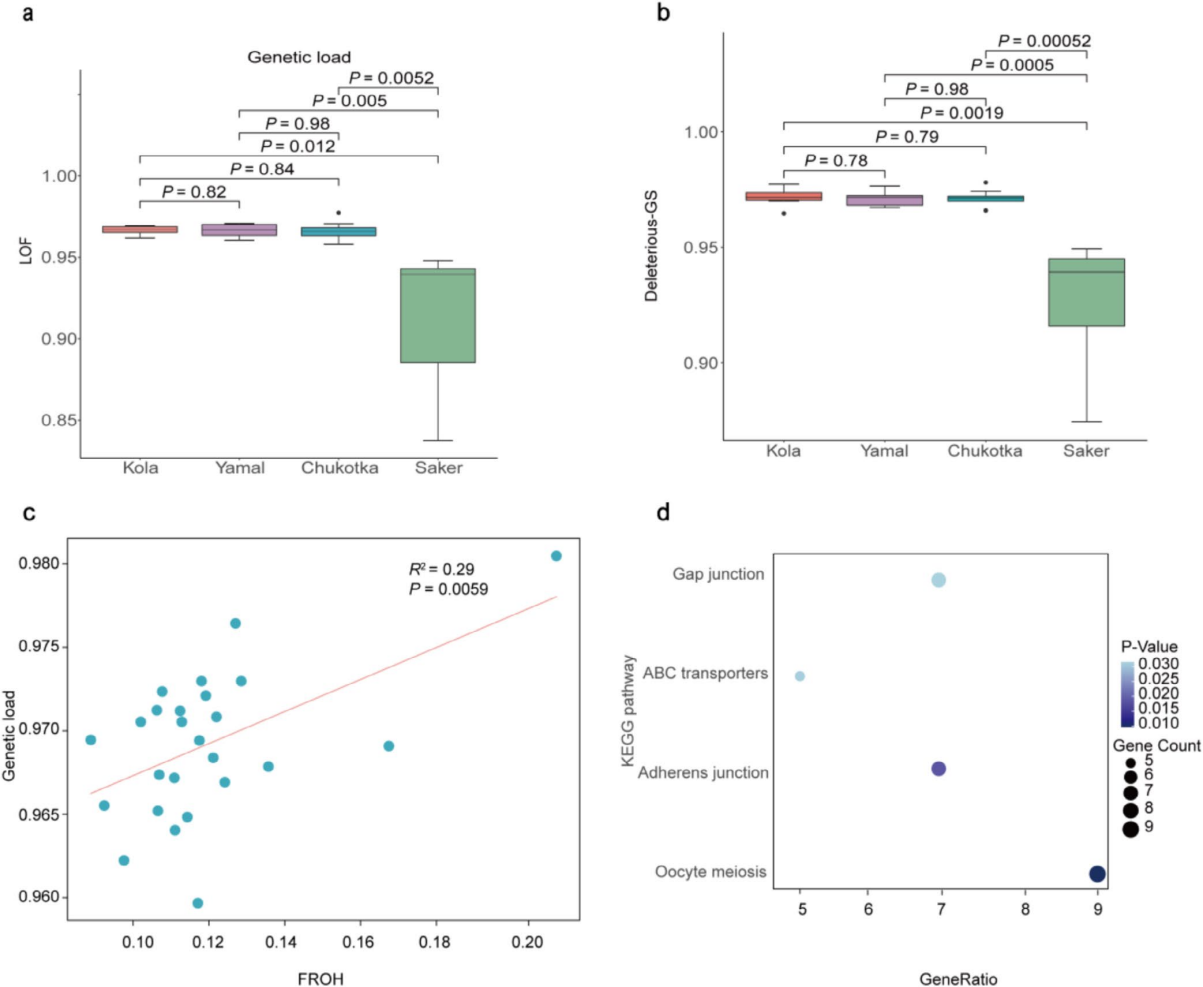
Genetic load comparisons among three gyrfalcon populations. (a, b) Genetic load of three gyrfalcon populations compared with saker falcon using loss‐of‐function (LOF) and deleterious‐GS (deleterious Grantham score < 150) respectively. (c) Correlation between genomic inbreeding coefficient based on run of homozygosity (FROH) and genetic load. (d) The KEGG pathways enriched from the genes with LOF variants.

Furthermore, our analysis of loss‐of‐function (LOF) and missense variants showed that the ratios of homozygous LOF and missense sites to total homozygous and heterozygous sites were comparable among the three gyrfalcon populations (Figure 4a,b). In contrast, these ratios were consistently higher in gyrfalcons than in sakers, indicating a greater relative burden of potentially deleterious variants in gyrfalcons. In addition, we also found a positive correlation between FROH and LOF genetic load in the three populations, indicating that high inbreeding level is a possible reason for high genetic load observed in the studied gyrfalcons (Figure 4c). As for genes affected by the homozygous putative deleterious LOF mutations in the gyrfalcon population, we found the LOF variants were significantly enriched in KEGG pathways associated with growth and disease (Figure 4d and Table S14).

### 3.6 Positively Selected Signatures Between Kola and Chukotka Populations

We identified 30 and 35 positively selected genes in the Chukotka and Kola population, respectively from the XP‐EHH analysis (Figure S7a; Tables S15 and S16). In the Kola population, *NFATC1* and *CDA* (Figure S7b) are genes involved in immune regulation and chemical metabolism. While in the Chukotka population, genomes were enriched in DNA repair genes, such as *FANCA* (interstrand crosslink repair) and *RBBP8* (DNA repair), respectively.

### 3.7 Population and Climatically Suitable Area Changes Under Future Warming

**FIGURE 5:**
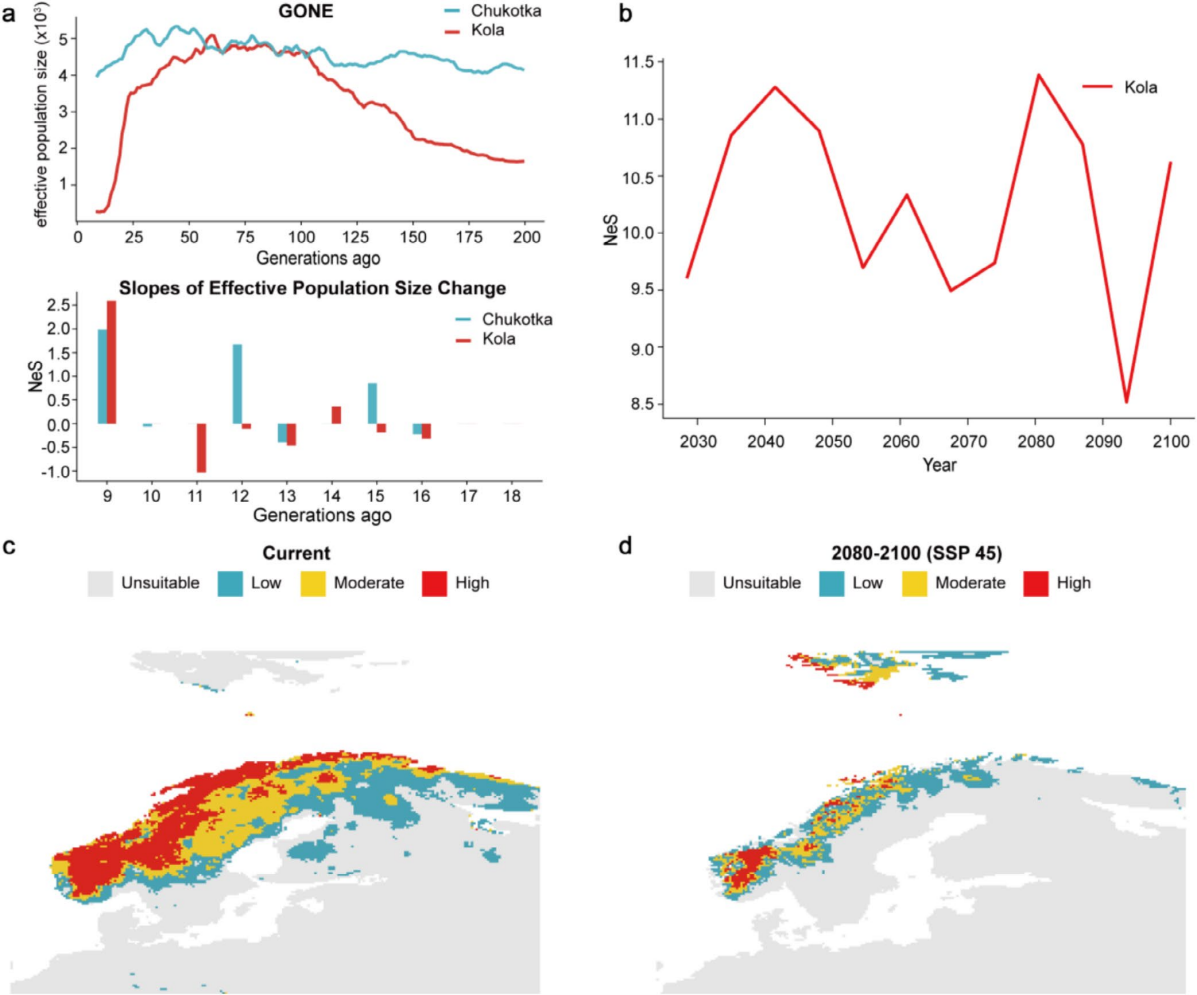
Prediction of climatically suitable areas for gyrfalcons under climate change. (a) Recent *N*e changes inference using GONE, and estimation of NeS for the Kola and Chukotka populations over the past 9–18 generations. (b) Prediction of NeS change for the Kola population (2028–2100) under the SSP245 scenario. (c, d) Prediction of climatically suitable areas for Eurasian Arctic gyrfalcons in present and 2080–2100 under the SSP245 scenario, respectively. Scores under “moderate” and “high” are considered as climatically suitable areas.

Our GONE analysis further revealed that the *N*e of Kola population underwent rapid decline during the past 25–9 generations (about 165–60 years) ago, while the Chukotka population slightly declined (Figure 5a). Generalized linear model (GLM) analysis showed that in Kola, that *N*_e_S was significantly negatively affected by extreme low temperature days and annual average temperature (*R* ^2^ = 0.9437, *p* = 0.018; Table S17), suggesting that climate is a driver for Kola population decline. In contrast, there is no obvious correlation between climate change and *N*_e_S fluctuation in the Chukotka population (Table S17).

Based on the current *N*_e_S estimation, our GLM model predicts a continued decline in effective population size for the Kola populations from 2028 to 2100 (Figure 5b; Figure S8a; Table S18). Furthermore, according to the SDM, the bioclimatic variables bio1 (mean annual air temperature), with average importance percentages of 62.31%, underscore the crucial role of temperature in shaping the distribution of gyrfalcon (Table S19). Overall, SDM projections indicate that by 2100, climatically suitable areas for gyrfalcon in the Kola will shift markedly northward and decline by 66.7% under the SSP245 scenario and by more than 90% under the SSP585 scenario (Figure 5c; Figure S8b; Table S20).

## 4 Discussion

### 4.1 Eurasian Gyrfalcon Population Divergence due to Climate Change

In this study, we have utilized 26 gyrfalcon genomes from three breeding regions of the Eurasian Arctic (i.e., Kola, Yamal and Chukotka) to study how climate change influences the population divergence of Eurasian gyrfalcons. Our analyses of population structure and population history dynamics indicate a clear east–west genetic differentiation among Eurasian gyrfalcon populations, spanning from the Kola population in Lapland to the Russian Far East (Chukotka population). This differentiation likely reflects historical processes acting over long evolutionary timescales. This divergence is estimated to have occurred approximately 14 ka, during the late Pleistocene epoch (Figure 2c,d). Population divergence following the Younger Dryas, coincides with the disappearance of the mammoth steppe‐tundra, which either resulted in isolation by distance along a circumpolar tundra distribution or by isolation due to steppe‐tundra fragmentation. Such patterns (i.e., glaciations driving regional isolation and population divergence) were also observed in Arctic vertebrates, for example in collared lemmings across northern Eurasia (Fedorov et al. 2020).

However, our study has limitations due to a lack of Nearctic gyrfalcon samples. Johnson et al. (2007) used eight microsatellite markers and did not identify any population genetic differentiation between the western Eurasian population (including Kola) and those of the continental Nearctic (only noted divergence between island populations of Greenland and Iceland with those of continental populations in North America and Lapland). The Chukotka population of eastern Eurasia may represent an extension of the Nearctic population, possibly contributing to the observation of east–west divergence across Eurasia identified in our genome‐level population analyses.

### 4.2 Historical Gene Flow Among Eurasian Gyrfalcon Populations and Recent Bottlenecks

Our *D*‐statistics analysis and fastsimcoal2 simulation have disclosed evidence of gene flow from Kola and Chukotka populations to the Yamal population in about 1.9 ka (Figure 2a,d). We hypothesize that this pattern is attributable to shifts in suitable habitats driven by climatic fluctuations, specifically the alternating warm and cold periods characteristic of the late Holocene. The Kola and Chukotka gyrfalcons may have expanded west‐east and contacted the Yamal population. Furthermore, our findings indicate the occurrence of a recent population bottleneck approximately 1 ka, coinciding with the Medieval Climate Anomaly, which stands as a significant factor contributing to the observed reduction in genome‐wide heterozygosity (Figures 2c and 3a). Hunting and trading in gyrfalcons, prevalent in Arctic Eurasia during the Middle Ages, may have also contributed to a population bottleneck during the Medieval Climate Anomaly (Shergalin 2011; Buquet 2021).

Historical climate change has profoundly shaped the demographic and ecological dynamics of Arctic‐breeding birds, influencing population sizes, habitat availability, and distribution patterns (Anderson et al. 2023; Patterson et al. 2025). Our genomic analyses provide evidence of gyrfalcon responses to past climatic shifts, revealing genetic signatures of gene flow and demographic fluctuations linked to environmental variability. These findings underscore the sensitivity of Arctic avifauna to climatic fluctuations and suggest that their historical capacity for evolution may confer resilience to ongoing climate change. However, the capacity of gyrfalcon response to climate change could be compromised if anthropogenic‐driven warming proceeds at a pace or magnitude that exceeds historical precedents, limiting opportunities for evolutionary or behavioral adjustment.

### 4.3 Arctic Warming Risks to the Genetically Vulnerable Gyrfalcons

In recent decades, the Arctic has been subject to warming significantly faster than the rest of the world, leading to profound changes in ecosystems and threats to wildlife (Rantanen et al. 2022). It is important to estimate the evolutionary potential of Arctic animals in response to Arctic warming. We found a significantly lower genome‐wide heterozygosity, higher inbreeding coefficients and elevated genetic load in three gyrfalcon populations compared with other threatened species, e.g., the sister species—saker falcon (EN) (Figures 3a,d and 4a,b), and Siberian cranes (CR) (Chen et al. 2025). These genomic patterns are likely the consequence of a recent population bottleneck that occurred approximately 1 ka, as well as the prolonged period of low *N*e since about 100 ka (Figure 2b,c). Genomic heterozygosity of the gyrfalcon is lower than those of endangered species Siberian crane, Dalmatian pelican and Kea, but a little higher than that of crested ibis ( *Nipponia nippon* ) (Li et al. 2014). Species with lower genomic diversity may have a higher extinction risk (Chen et al. 2024). We also found higher genetic load in Eurasian gyrfalcons, of which the LOF variants were associated with disease (Table S14), such as avian influenza. Highly pathogenic avian influenza (HPAI) has been proposed as a contributing factor to recent gyrfalcon declines in Iceland and Alaska (Günther et al. 2022; Radcliffe et al. 2024). Nevertheless, the gyrfalcon is classified as Least Concern on the IUCN Red List due to their estimated relatively stable population size. Thus, the mismatch between low genetic diversity of gyrfalcon and its current conservation status indicates that the species could be more susceptible than expected to potential threats, including avian influenza.

As global warming accelerates, anticipating species' responses to future climate change is critical for prioritizing conservation efforts in the Arctic. Our GONE results suggested that the effective population size of gyrfalcon in the Kola Peninsula has declined over the past century (Figure 5a). Earlier estimates of a long‐term decline in Fennoscandia (Cramp and Simmons 1980) were criticized by Koskimies as exaggerations due to methodological flaws, and contemporary and historical records of the number of gyrfalcons in Northern Fennoscandia indicate the population has not sharply declined in the past 150 years (Koskimies 2011). One possible reason for this discrepancy is that we estimated the recent *N*e (not real *N*) based on LD methods (GONE and NeS), which might associate with the highest LD decay in the Kola population (lower recombination compared with Yamal and Chukotka populations) (Figure 3e).

The recent *N*e decline was inferred to be likely due to the effects of climate warming (Figure 5a). Gyrfalcons are highly specialized predators, with breeding success and population dynamics closely tied to grouse availability (Nielsen and Pétursson 1995; Nyström et al. 2006; Barraquand and Nielsen 2018). Thus, climate mediated effects on prey species and their habitats will also influence gyrfalcon populations (Gustafson et al. 2025). Furthermore, our SDM simulation results revealed that continued Arctic warming will drive a northward shift in the climatically suitable areas of gyrfalcons, probably reduce suitable habitat area, and increase survival pressure on Kola gyrfalcons. We note that these projections are based on climate model outputs, which inherently contain uncertainties. Although such models represent the best available estimates of future climate conditions, these uncertainties should be considered when interpreting predicted range shifts and demographic responses. Taken together with their genomic vulnerability, the declining trend in *N*e and range contraction under future warming, we thus call for conservation actions for the gyrfalcon.

### 4.4 Conservation of Arctic Animals Under Global Warming

Illegal trapping and trade in wild‐caught gyrfalcons is an on‐going conservation threat (Wyatt 2011), where successful law enforcement often results in the acquisition of confiscated gyrfalcons of unknown Eurasian Arctic origin (Lobkov et al. 2011). The low level of genetic differentiation across the continental breeding distribution of gyrfalcons in Eurasia and the Nearctic reported by Johnson et al. (2007), agrees with our more detailed genomic analysis of the Eurasian population, where limited differentiation is clinal. Consequently, the release of gyrfalcons that have been confiscated from trappers in the intermediate population region of the central Siberian Arctic (e.g., Yamal) does not require specific knowledge of their Eurasian population origin and is compatible with IUCN genetic considerations for releases (IUCN/SSC 2013). For gyrfalcons and other Arctic‐adapted animals, conservation efforts should prioritize maintaining connectivity among isolated populations and monitoring the population genetic diversity.

Our study provides a comprehensive assessment of the spatiotemporal evolutionary and ecological responses of a resident Arctic raptor to past, present and future climate fluctuations. Increasing isolation and contraction of habitats are expected to reduce gene flow between populations, elevate inbreeding, and ultimately erode both genetic integrity and adaptive potential. The future resilience of Arctic predators like the gyrfalcon will depend not only on their ecological tolerance but also on proactive conservation strategies. More generally, our findings highlight the power of combining whole‐genome sequencing, demography inference, and ecological modeling in studying reconstructing population histories, estimating current genetic diversity and forecasting future vulnerability for Arctic species. By linking genomic signatures of divergence and gene flow to climate‐driven habitat change, we provide a framework for assessing the evolutionary potential of Arctic wildlife in a rapidly changing world. These integrative approaches are essential for developing evidence‐based conservation strategies considering both historical contexts and future ecological uncertainties.

## Author Contributions

**Xin Liu:** formal analysis (equal), writing – original draft (equal), writing – review and editing (equal). **Li Hu:** formal analysis (equal), writing – original draft (equal), writing – review and editing (equal). **Zhenzhen Lin:** data curation (equal). **Shengkai Pan:** formal analysis (equal). **Siying Huang:** data curation (equal). **Vasiliy Sokolov:** funding acquisition (equal), investigation (equal). **Aleksandr Sokolov:** investigation (equal). **Ivan Fufachev:** investigation (equal). **Sergey Ganusevich:** investigation (equal). **Andrew Dixon:** conceptualization (equal), project administration (equal), writing – original draft (supporting), writing – review and editing (equal). **Xiangjiang Zhan:** conceptualization (lead), project administration (lead), writing – original draft (lead), writing – review and editing (lead).

## Funding

This study was supported by National Natural Science Foundation of China grants 32361133559 and 32125005 (to X.Z.), 32370460 (to L.H.), Russian Science Foundation grant 24‐44‐00094 which also cover partial sampling in Yamal (to A.S., V.S. and I.F.), International Partnership Program of the Chinese Academy of Sciences for Grand Challenges 073GJHZ2023091GC (to X.Z.), Third Xinjiang Scientific Expedition and Research Program grant 2021XJKK0600 (to Z.G.), Taishan Scholars Program of Shandong Province grant tsqn202312276 (to L.H.). Gyrfalcon sampling across Eurasia and partial genomic resequencing was funded by the Environment Agency‐Abu Dhabi.

## Conflicts of Interest

The authors declare no conflicts of interest.

## Supporting information


**Data S1:** ece373052‐sup‐0001‐Supplementary file.docx.

**Data S2:** ece373052‐sup‐0002‐Supplementary Table.xlsx.

## Data Availability

PacBio, HiSeq data for genome assembly, whole‐genome resequencing data for 18 gyrfalcon individuals and vcf file generated in this study have been deposited in the GSA database under accession code CRA031960 (BioProject code: PRJCA048966). The gyrfalcon genome assembly has been deposited in the GWH database under accession code GWHGSGT00000000.1. Whole‐genome resequencing data for 8 gyrfalcons and 7 saker falcons used in this study are available in the GSA database under accession code CRA00383 (BioProject code: PRJCA010321). The code used in this study is available at https://doi.org/10.5281/zenodo.18409074.
