# Supplementary material for: Evolution and Climate Adaptation in Eurasian Gyrfalcon Populations
Source: Ecol Evol. 2026 Feb 10;16(2):e73052. doi: 10.1002/ece3.73052 (PMC12891816; doi:10.1002/ece3.73052)
Supplement: Supplementary file 1 — Data S1: ece373052‐sup‐0001‐Supplementary file.docx. [file ECE3-16-e73052-s002.docx]

**Supplemental Information for:**

**Evolution and Climate Adaptation in Eurasian Gyrfalcon Populations**

**Supplementary Methods**

**Habitat Suitability Classification**

SDMs were performed using the ensemble modelling approach implemented in the R package biomod2 (version 4.2-6), incorporating eight algorithms. We generated two independent sets of 1,000 pseudo-absence points by randomly sampling from the background. The performance of each model was evaluated using the True Skill Statistic (TSS) (Allouche et al. 2006) and the Area Under the Receiver Operating Characteristic Curve (AUC) (Hanley and Mcneil 1982). Models meeting the criteria of TSS > 0.6 and AUC > 0.8 were retained for ensemble construction. Ensemble predictions were then derived by calculating a TSS-weighted mean of the probabilities across the selected models.

To assess habitat suitability for gyrfalcons under past, present, and future climate scenarios, predicted suitability scores (0-1) were rescaled using max–min normalization in *R*, ensuring comparability across regions and time periods. The normalized values were then classified into four categories: Unsuitable habitat: normalized suitability score < 0. 3, poor suitable habitat: 0.3 ≤ score < 0.6, moderate suitable habitat: 0.6 ≤ score < 0.8, high suitable habitat: score ≥ 0.8. These thresholds follow commonly used classification schemes in species distribution modeling, balancing sensitivity and specificity. This framework was applied consistently across past, present, and predicted climatically suitable areas under two climate scenarios (SSP245 and SSP585) in 2080 - 2100, enabling robust spatial comparisons and the detection of potential range shifts and habitat fragmentation. In addition, climatically suitable area changes under future climate conditions were quantified using the BIOMOD_RangeSize function. The relative importance of environmental predictors across algorithms and datasets was evaluated using the variables.importance function in biomod2 (**Table S19**).

**Prediction of effective population size changes in the future**

We constructed a GLM to model the relationships between *N_e_S* and climate variables. The generation time of gyrfalcons is estimated at 6.6 years; however, we used 6.5 years in the GLM for computational convenience when aligning with climate predictors (e.g., extreme high temperature frequency, mean temperature):

$N_{e}S_{t}$ = $a$ + $b_{t}*med \left\{ {TAV}_{i-6.5}^{i} \right\}$ + $c_{t}*min \left\{ {TMIN}_{i-6.5}^{i} \right\} {+ d}_{t}*max\left\{ {TMAX}_{i-6.5}^{i} \right\}$ + $\varepsilon_{t}$

**Supplementary Figures**

**
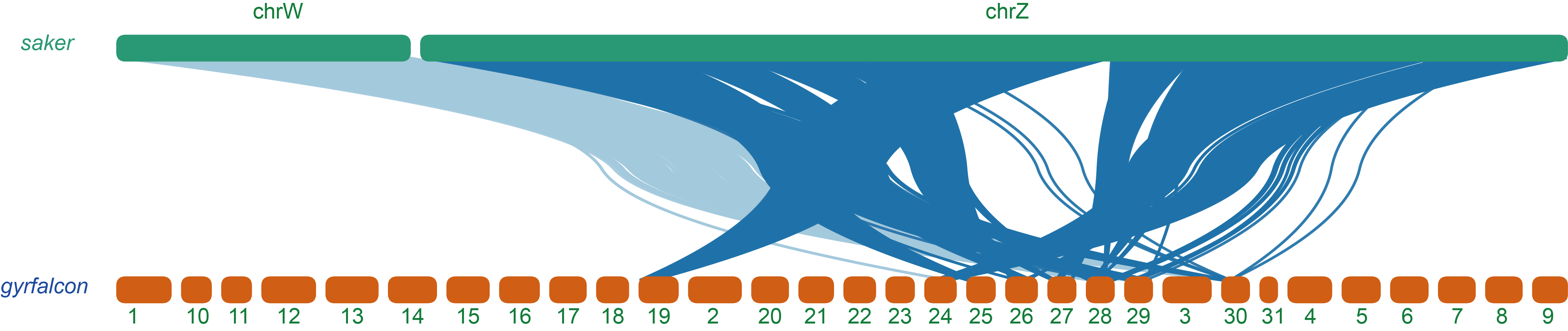
**

**Figure S1. Collinearity between super-scaffolds of gyrfalcon genome and sex chromosomes of saker falcon**.


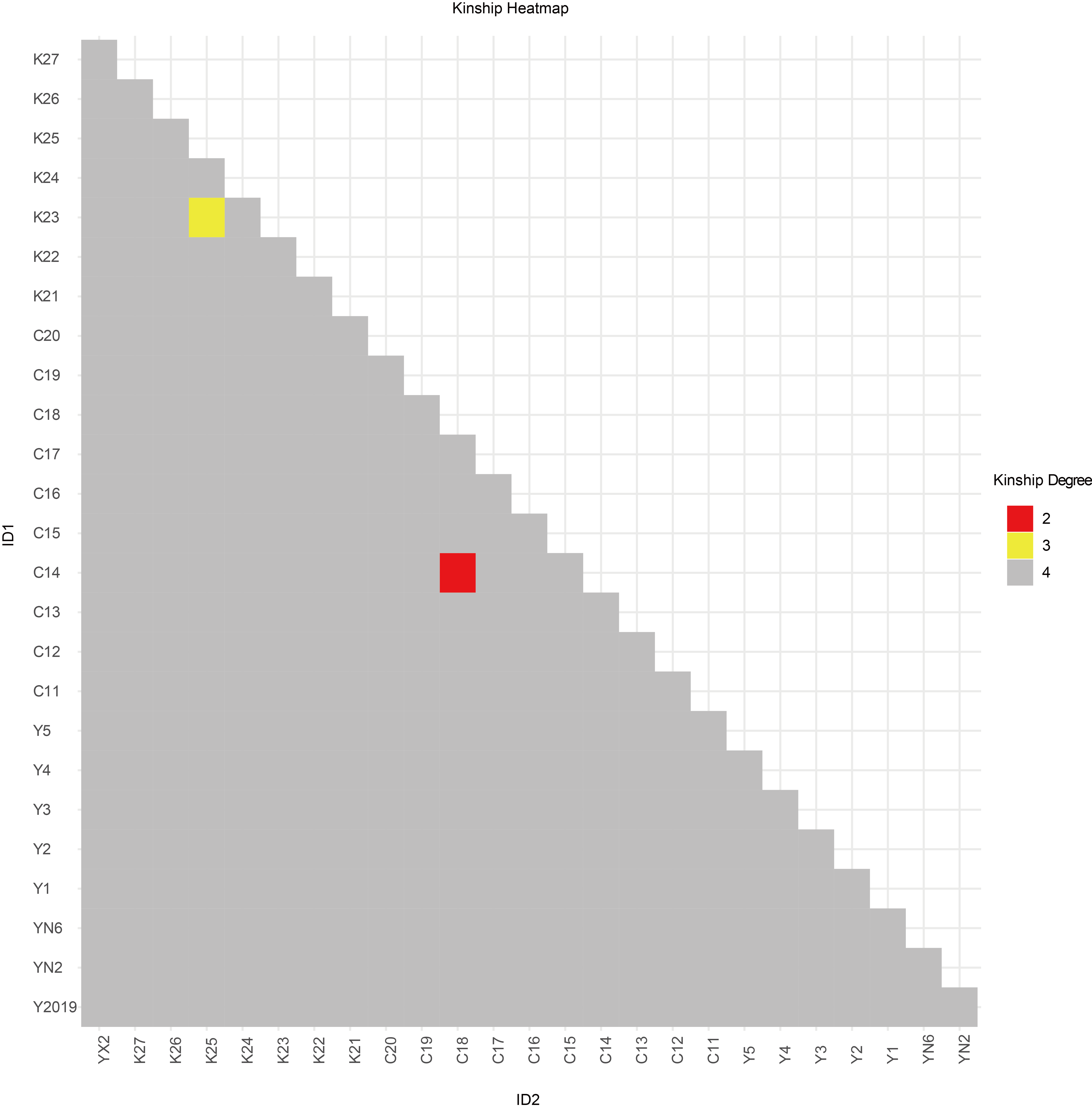


**Figure S2**. **Kinship of all gyrfalcon individuals**. The relationships between gyrfalcon pairs were determined as first-order kinship (kinship value ≥ 0.177; includes parent-child relationships as well as full siblings), second-order kinship (red blocks; 0.0884 ≤ kinship value < 0.177; half-siblings which share one parent but not both, as well as grandparent-grandchild relationships), third-order kinship (yellow blocks; 0.0442 ≤ kinship value < 0.0884; includes cousins), and unrelated (grey blocks; kinship value < 0.0442).
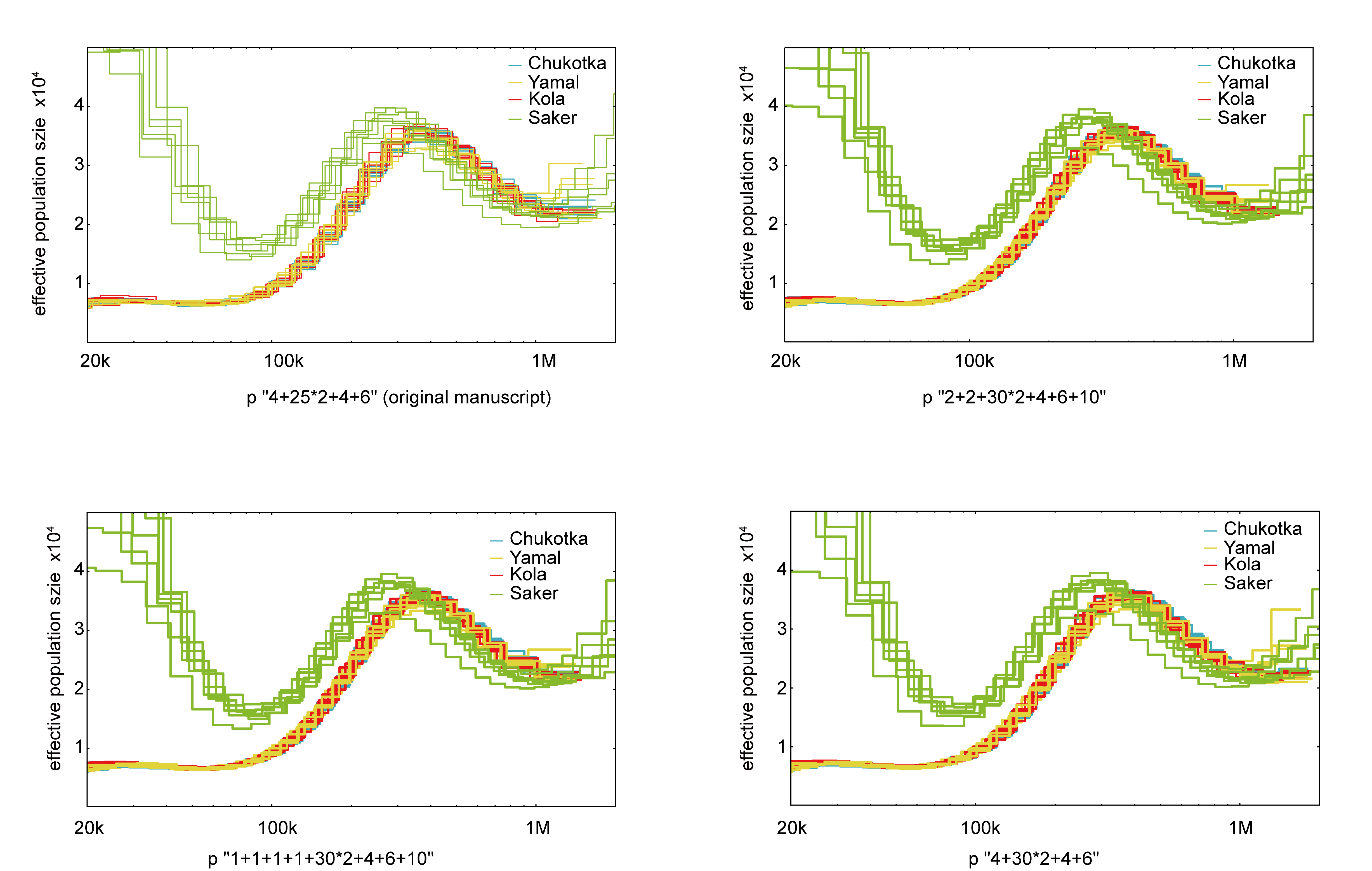


**Figure S3. PSMC results using different parameters, -p*.***


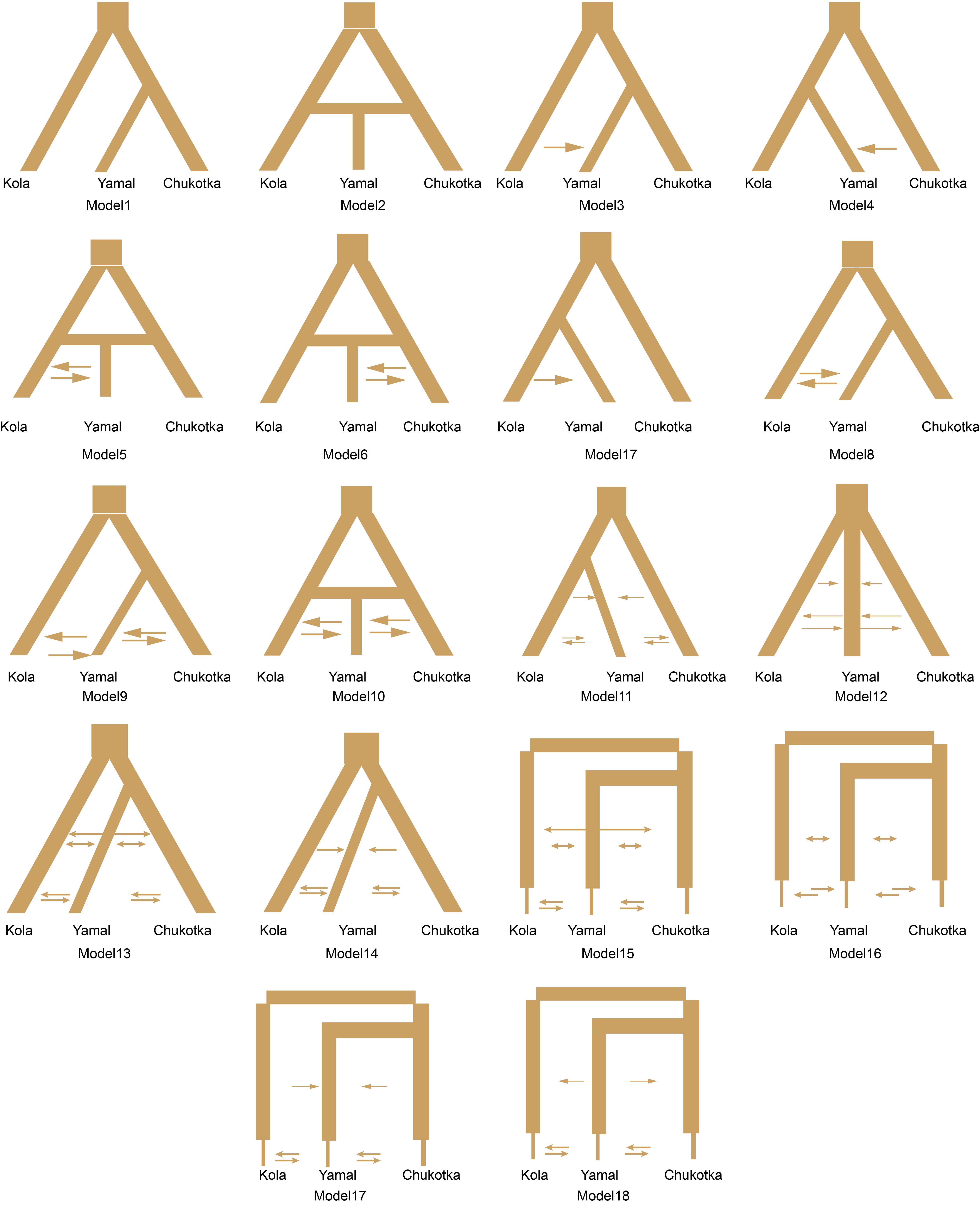


**Figure S4. Eighteen simulated demographic historical models among gyrfalcon populations using *fastsimcoal2.*** Arrows indicate directions of gene flow events between populations.


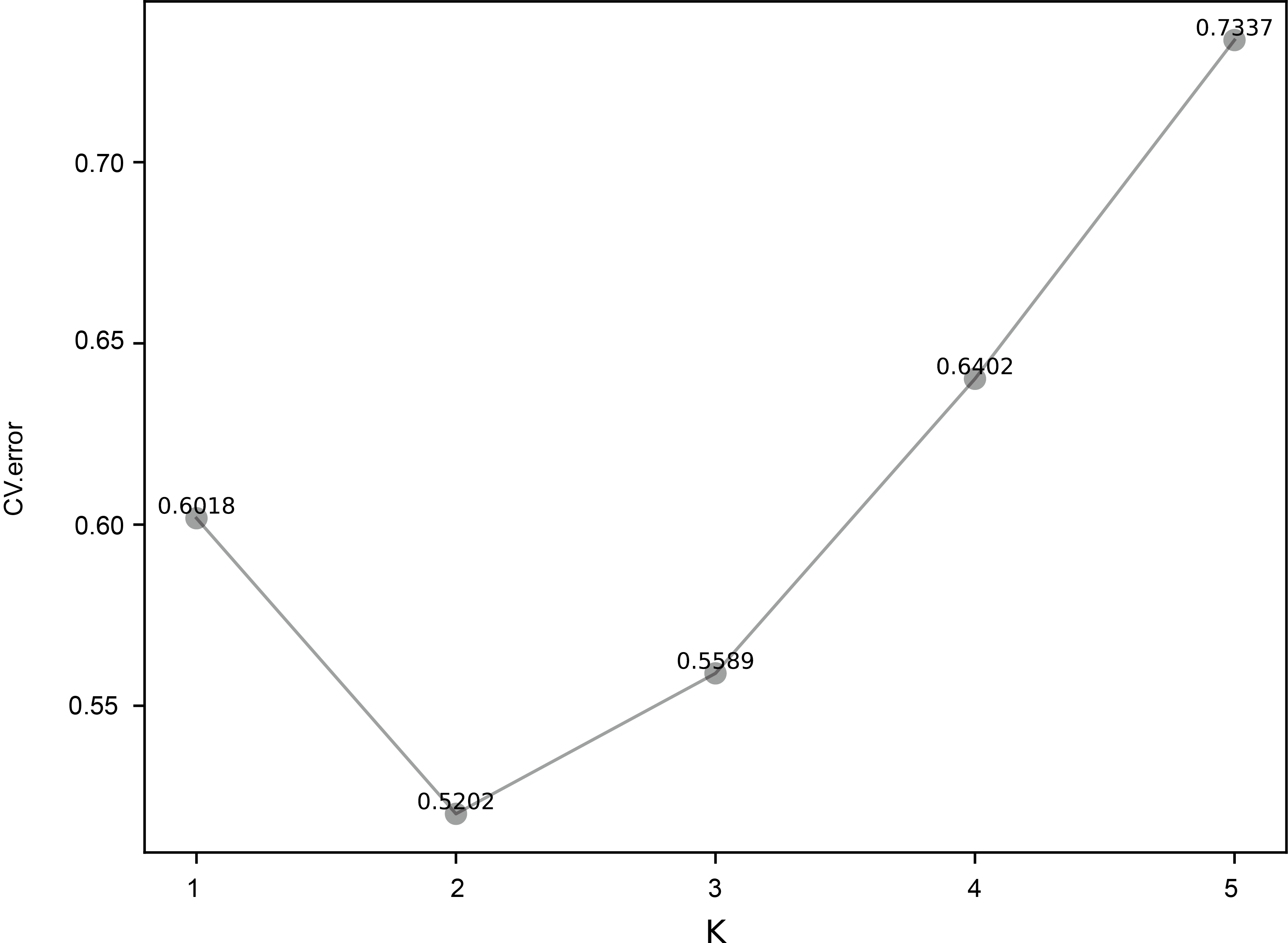


**Figure S5**. **The cross validation (CV) errors for various *K* values (1–5) in ADMIXTURE analysis**.


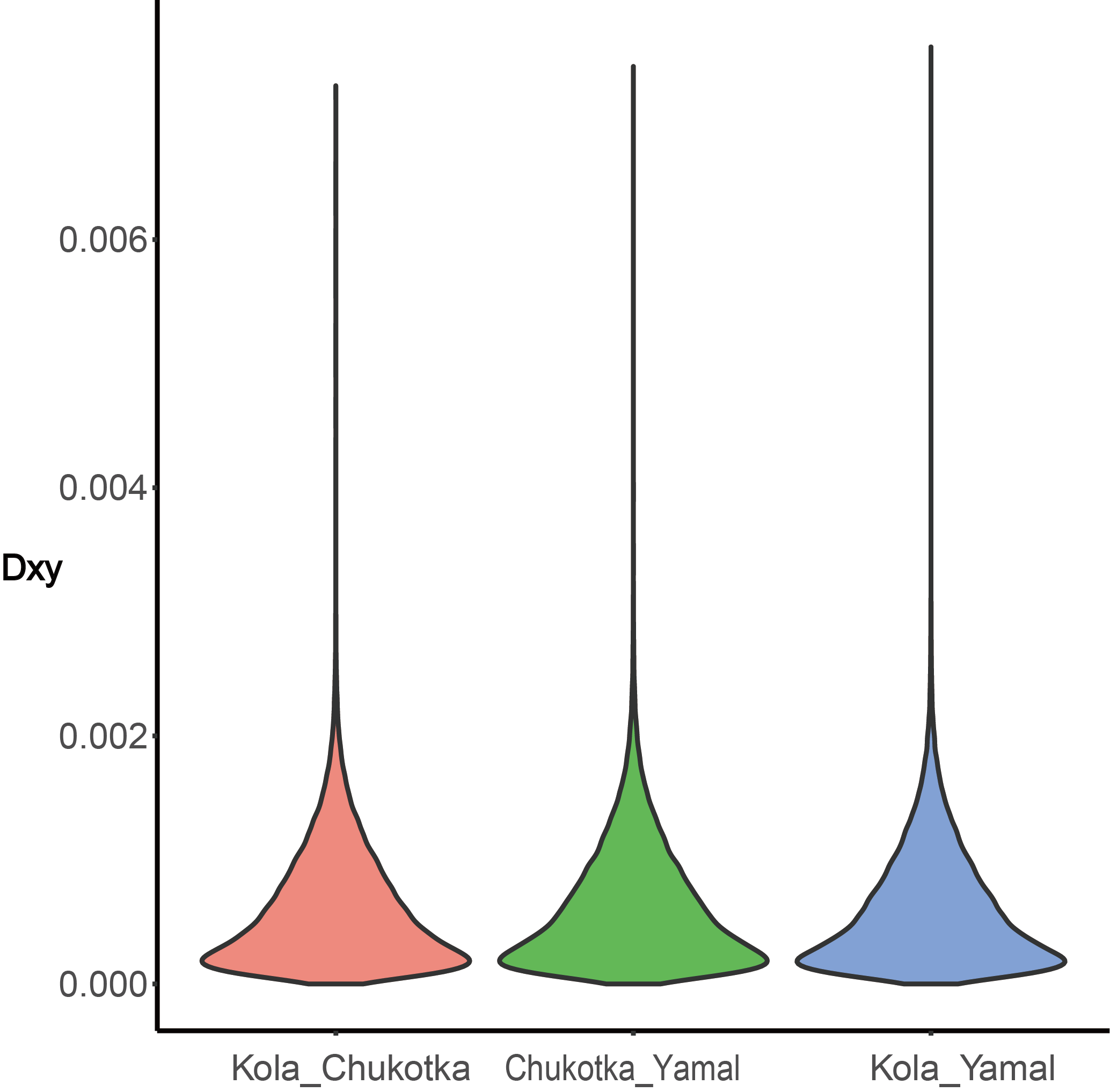


**Figure S6. Pairwise *d*_XY_ values (Kola-Yamal; Chukotka-Yamal; Kola-Chukotka)**


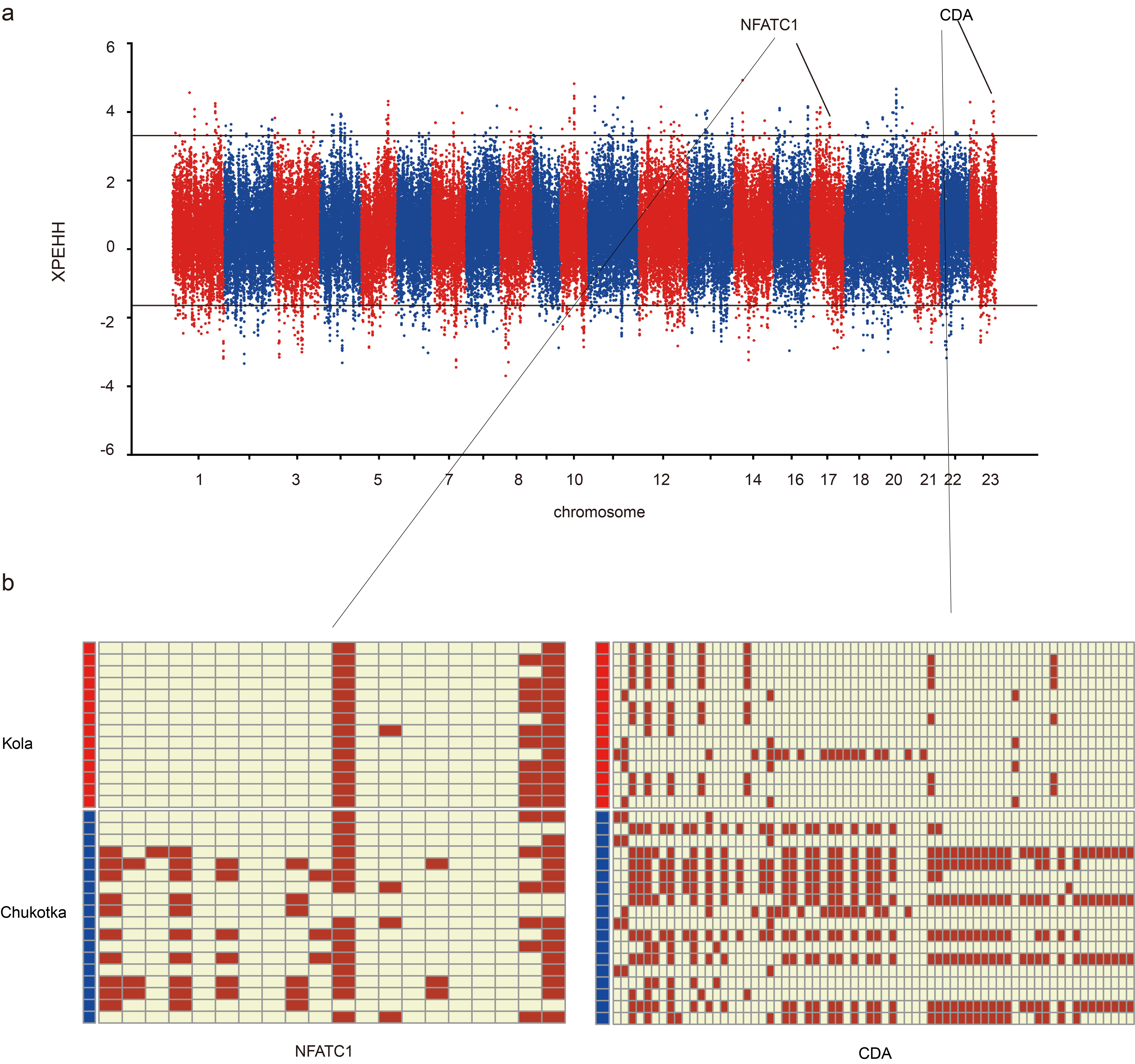


**Figure S7.** (a) Manhattan plot showing the estimated XP-EHH values for each 10-kb window sliding along genome between the Kola and Chukotka population. The top and bottom lines represent selected regions in Kola population (top 1%) and Chukotka population (bottom 1%) respectively. (b) Haplotypes of the focal *NFATC1* and *CDA* in gyrfalcons.


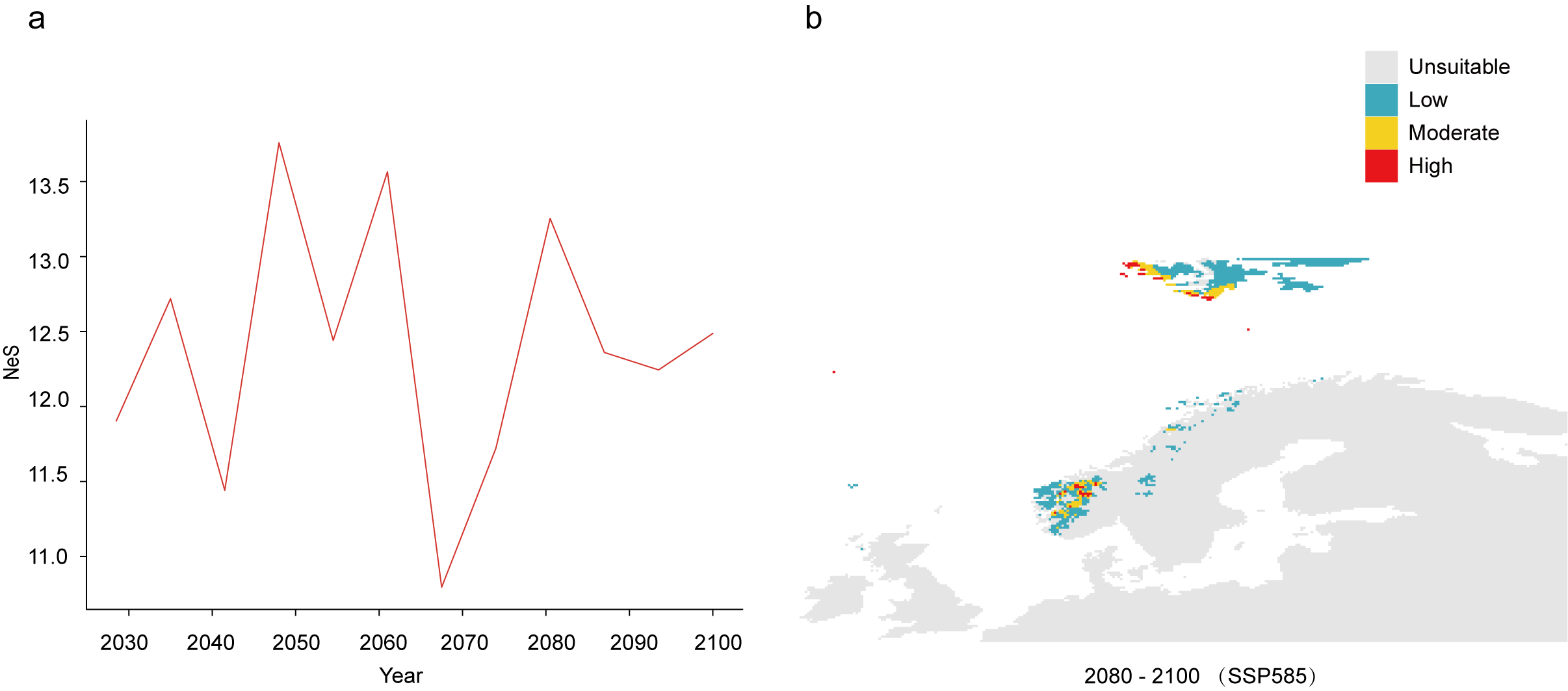


**Figure** **S8.** (a) Projected changes in *N*eS for the Kola population (2028-2100) under the SSP585 scenario. (b) Eurasian distribution of Kola population in 2080-2100 under the SSP585 scenario. Scores under “moderate” and “high” are considered as suitable habitats.

**Reference**

Allouche, O., et al. (2006). “Assessing the Accuracy of Species Distribution Models: Prevalence, Kappa and The True Skill Statistic (TSS).” *Journal of Applied Ecology* **43**, no. 6: 1223-1232.

Hanley, J. A. and B. Mcneil (1982). “The Meaning and Use of the Area Under a Receiver Operating Characteristic (ROC) Curve.” *Radiology* **143**, no. 1: 29-36.
